# Correlates of Pain, Opioid Use, and Psychotropic Medications Among Older African Americans

**DOI:** 10.1093/geroni/igaa057.2844

**Published:** 2020-12-16

**Authors:** Sharon Cobb, Mohsen Bazargan, Shervin Assari

**Affiliations:** Charles R. Drew University of Medicine & Science, Los Angeles, California, United States

## Abstract

Unrecognized and undertreated pain among older African Americans (AAs) is well-documented. This study explored the link between social, behavioral, and health correlates of pain and psychotropic as well as opioid-based medications in a sample of underserved 740 AA older adults. Almost 16% and 17% of participants used at least one psychotropic and opioid-based medications, respectively. Of those who use opioid-based medications, 73% used opioids only, 28% used opioids and at least one psychotropic medication. Use of opioid or psychotropic medications were associated with increased polypharmacy. Multivariate analysis showed different types of pain are predictors of opioid use, however, depressive symptoms and level of pain were associated with use of psychotropic medication. Moreover, the relationship of pain and psychotropic medications warrants more study due to emerging mental health crisis. These findings underscore the need for optimal concurrent management of pain and mental health for older AAs with potential inappropriate medication use.

